# Profiles and correlates of language and social communication differences among young autistic children

**DOI:** 10.3389/fpsyg.2022.936392

**Published:** 2022-09-06

**Authors:** Rachel Reetzke, Vini Singh, Ji Su Hong, Calliope B. Holingue, Luther G. Kalb, Natasha N. Ludwig, Deepa Menon, Danika L. Pfeiffer, Rebecca J. Landa

**Affiliations:** ^1^Center for Autism and Related Disorders, Kennedy Krieger Institute, Baltimore, MD, United States; ^2^Department of Psychiatry and Behavioral Sciences, The Johns Hopkins University School of Medicine, Baltimore, MD, United States; ^3^Department of Mental Health, Johns Hopkins Bloomberg School of Public Health, Baltimore, MD, United States; ^4^Department of Neuropsychology, Kennedy Krieger Institute, Baltimore, MD, United States

**Keywords:** autism, latent profile analysis, correlates, child-based factors, sociodemographic factors

## Abstract

Delays in early language development are characteristic of young autistic children, and one of the most recognizable first concerns that motivate parents to seek a diagnostic evaluation for their child. Although early language abilities are one of the strongest predictors of long-term outcomes, there is still much to be understood about the role of language impairment in the heterogeneous phenotypic presentation of autism. Using a person-centered, Latent Profile Analysis, we first aimed to identify distinct patterns of language and social communication ability in a clinic-based sample of 498 autistic children, ranging in age from 18 to 60 months (*M* = 33 mo, *SD* = 12 mo). Next, a multinomial logistic regression analysis was implemented to examine sociodemographic and child-based developmental differences among the identified language and social communication profiles. Three clinically meaningful profiles were identified from parent-rated and clinician-administered measures: Profile 1 (48% of the sample) “Relatively Low Language and Social Communication Abilities,” Profile 2 (34% of the sample) “Relatively Elevated Language and Social Communication Abilities,” and Profile 3 (18% of the sample) “Informant Discrepant Language and Relatively Elevated Social Communication Abilities.” Overall, young autistic children from the lowest-resource households exhibited the lowest language and social communication abilities, and the lowest non-verbal problem-solving and fine-motor skills, along with more features of attention-deficit/hyperactivity disorder and atypical auditory processing. These findings highlight the need for effective community-based implementation strategies for young autistic children from low-resource households and underrepresented communities to improve access to individualized quality care.

## Introduction

Autism is one of the most common and complex neurodevelopmental conditions, with an early-onset (Landa and Garrett-Mayer, 2006; Ozonoff et al., 2010) high heritability (heritability of 0.9; Tick et al., 2016), and increasing prevalence (1 in 44; Maenner, 2021). Language delays are characteristic of young autistic children (Landa and Garrett-Mayer, 2006; Ozonoff et al., 2010; Tager-Flusberg, 2016), and one of the most recognizable first concerns that motivates parents to seek a diagnostic evaluation for their child (Herlihy et al., 2015; Talbott et al., 2015). Although early language abilities (prior to age 6 years) are one of the strongest predictors of later academic performance, relationships, and quality of life (Petersen et al., 2013; Howlin and Magiati, 2017), there is still much to be understood about the role of language impairment in the heterogeneous phenotypic presentation of autism.

Just as autistic children exhibit significant heterogeneity across all levels of the phenotype (Jeste and Geschwind, 2014), they also show a range of language profiles. Studies have revealed that variability in language development is most pronounced prior to age 6 (Landa et al., 2013; Randall et al., 2016; Brignell et al., 2019) with more stable language profiles observed from prior to age 6 to 19 years (Pickles et al., 2014). Although many children develop spoken language, despite delays early in life, approximately 30% of autistic children use no to fewer than 20–30 spoken words beyond age 5 (Tager-Flusberg and Kasari, 2013; Tager-Flusberg, 2016). Differences in the use and understanding of language have been found to vary greatly (Ellis Weismer et al., 2010; Landa et al., 2013; Pickles et al., 2014; Whyte and Nelson, 2015). Some studies report greater difficulty in receptive language relative to expressive language (Charman et al., 2003; Luyster et al., 2008; Hudry et al., 2010), while others have found the opposite pattern (Luyster et al., 2008; Ellis Weismer et al., 2010; Kover et al., 2013). In support of the variability in receptive-expressive language profiles observed across studies, a meta-analysis examined discrepancies in receptive and expressive language ability among younger (Age: 1 to 5 years) and older (Age: 6 to 19 years) groups of autistic children and youth, and did not find evidence of a common receptive-expressive language profile in either age group (Kwok et al., 2015).

Social communication and interaction skills (hereafter, social communication) are intimately linked with language development in young autistic children. Based on the DSM-5 criteria, social communication skills broadly encompass behaviors related to social-emotional reciprocity (e.g., reduced sharing of emotions/affect/interests and difficulty initiating/responding to others), non-verbal communication (e.g., difficulty using and understanding gestures/body postures), and developing and maintaining relationships (e.g., lack of interest in others, difficulty making friends, and limited imaginative play) (American Psychiatric Association, 2013). Extant evidence suggests that there may be a bidirectional relationship between social communication and language development such that early social communication skills (i.e., initiation of and response to joint attention, gesture use, and imitation) are predictive of spoken language outcomes (Yoder et al., 2015; Delehanty et al., 2018; Pecukonis et al., 2019); and early expressive language skills are predictive of social communication outcomes (Anderson et al., 2009; Dillon et al., 2021). This can be seen in a retrospective study that examined predictors of language outcomes in a sample of 535, 8-year-old autistic children with significant language delay from the Simons Simplex Collection (Wodka et al., 2013). This study reported that higher levels of parent-reported social communication skills were associated with the acquisition of phrase and fluent speech, as well as an earlier age of language acquisition (even when non-verbal cognition was taken into account; Wodka et al., 2013). In contrast, other studies have found that social communication is not strongly associated with language development after other factors, such as non-verbal cognition, are considered (Sigman and McGovern, 2005; Thurm et al., 2015). One reason for the discrepancy in findings across studies may be due to differences in sample size and, as a result, statistical power. For example, Sigman and McGovern, 2005’s study included 48 participants; Thurm et al., 2015’s study included 70 participants; whereas Wodka et al., 2013’s study included 535 participants. Another reason for the discrepancy in findings may be due to differences in sample sociodemographic factors (e.g., child age, sex, race, parental education) and/or child-based characteristics (e.g., developmental functioning, presence of co-occurring conditions). Given the heterogeneity of language and social communication profiles observed across young autistic children, it is important to identify factors that may account for such variability to improve the development of and access to individually tailored interventions aimed at improving language and social communication outcomes.

In terms of sociodemographic factors, lower parental education is one of the most consistently reported correlates of lower language abilities in autistic children (Anderson et al., 2007; Pungello et al., 2009; Warlaumont et al., 2014; Ellis Weismer and Kover, 2015; Olson et al., 2021). For example, Olson et al. (2021) examined the association between household- and neighborhood-level socioeconomic status variables and receptive and expressive language skills in autistic and neurotypical 15- to 64-month-olds. Lower maternal education was more strongly associated with lower receptive and expressive language skills across both participant groups, compared to income-based socioeconomic variables (Olson et al., 2021). Similarly, Ellis Weismer and Kover (2015) found that maternal education contributed to the correct classification of 80% of autistic children into high versus low language groups at age 5.5 years.

To date, no direct relationship between race and language development has been reported in the autism literature. However, there is evidence to suggest an indirect link between child/family race and language, and social communication outcomes. Recent reports indicate that children from underrepresented racial groups are less likely to receive an ASD evaluation compared to their non-Hispanic White counterparts by the child’s third birthday (Maenner, 2021), which inevitably leads to delays in access to intervention services. Such intervention delays could hold negative consequences for language and social communication development, as history of intervention services has been associated with positive language and social communication outcomes (Mazurek et al., 2012).

Among child-based developmental factors, non-verbal cognition is one of the most consistently reported correlates of language outcomes (Mazurek et al., 2012; Wodka et al., 2013; Ellis Weismer and Kover, 2015; Thurm et al., 2015; Bal et al., 2020). Ellis Weismer and Kover (2015), found that non-verbal cognition at 2.5 years was a strong predictor of expressive language at 5.5 years. In two separate Simons Simplex Collection cohort studies, Mazurek et al. (2012) and Wodka et al. (2013) found that social communication intervention gains and acquisition of phrase or fluent speech were greatest among autistic children and adolescents with higher non-verbal cognition, respectively. Evaluating non-verbal cognition as a correlate of language outcome affords an estimation of general cognitive ability without the confound of language ability (DeThorne and Schaefer, 2004). As such, if a low level of non-verbal cognition is a correlate of similarly low levels of receptive and expressive language then such a profile may be indicative of a broad developmental delay, as opposed to a delay specific to language.

Beyond non-verbal cognition, auditory processing is another putative correlate of language outcomes in autism (Haesen et al., 2011; Boucher, 2012; Kujala et al., 2013; Matsuzaki et al., 2019). Here, one hypothesis is that difficulty in efficiently representing brief sounds and rapid auditory transitions (i.e., auditory temporal processing) may lead to further difficulty distinguishing phonemic contrasts (e.g., /ba/ vs. /ga/). Consistent with this hypothesis, children with lower language ability have shown difficulty in tasks that require the encoding of rapid spectrotemporal changes in auditory signals (Tallal and Gaab, 2006); and positive correlations have been found between auditory processing and later expressive language outcomes in infants at elevated likelihood for developing autism (Riva et al., 2018).

Both prospective (Bhat et al., 2012; LeBarton and Iverson, 2013; Iverson et al., 2019; LeBarton and Landa, 2019; Bal et al., 2020) and retrospective studies (Mody et al., 2017) have additionally shown a positive association between fine motor skills and later language and social communication development. Fine motor skills, the ability to coordinate the small muscles of the fingers and hands to reach, grasp, and manipulate objects, have been found to be more susceptible to delay in autism, when compared to gross motor skills, such as walking (Landa et al., 2012). This may explain some of the variability in language and social communication development among young autistic children, as evidence suggests that infants with more sophisticated object manipulation and exploration have increased opportunities to interact with their environment and learn from their caregivers. This in turn provides a rich scaffolding for language development (for a review see, Iverson, 2021).

Finally, features of other co-occurring neurodevelopmental and psychiatric conditions have also been associated with differences in language and social communication abilities among young autistic children. Indeed, autistic children with co-occurring attention deficit/hyperactivity disorder (ADHD) symptoms are often reported to have greater impairments in communication and socialization skills (Rao and Landa, 2014; Lyall et al., 2017; Yerys et al., 2019). Features of anxiety are also commonly observed in young autistic children and have been found to also interact with language and social communication abilities. However, unlike the inverse relationship observed between ADHD symptoms and language/social communication abilities, young autistic children with higher expressive language abilities tend to exhibit higher levels of anxiety symptoms, and vice versa (Davis et al., 2012; Vasa et al., 2016; Rodas et al., 2017).

To our knowledge, no study to date has characterized concurrent patterns of language *and* social communication abilities in a sample of autistic toddlers and preschoolers using a person-centered latent profile analytic (LPA) approach. LPA is particularly useful for describing the heterogeneity observed in a given sample by identifying subgroups (or latent profiles) with similar patterns of performance across multiple domains (Collins and Lanza, 2009; Lanza et al., 2013). In addition, no study has examined the extent to which sociodemographic and child-based developmental factors—commonly observed to interact with language and social communication development early in life—are associated with different profiles of language and social communication abilities among young autistic children. Identifying specific correlates of language and social communication profiles (beyond global non-verbal cognitive ability) is important to inform the development of individualized intervention targets (Bal et al., 2020; Saul and Norbury, 2020). To this end, we ask the following research questions: (1) How many qualitatively different profiles of language and social communication can be identified in a clinic-based sample of autistic toddlers and preschoolers using a person-centered (LPA) analytic approach?; (2) Are sociodemographic (i.e., race, parental education, medical insurance status, history of intervention) and child-based developmental factors (non-verbal problem solving skills, fine motor skills, auditory processing, as well as commonly co-occurring symptoms of ADHD and anxiety) differentially associated with identified language and social communication latent profiles?

## Materials and methods

### Participants

Data for this retrospective study were obtained from a sample of young autistic children who received a comprehensive autism spectrum disorder (ASD) evaluation at an urban, outpatient ASD specialty clinic located in the Mid-Atlantic region of the United States between June 2014 and December 2019. This research was approved by the Johns Hopkins Medicine Institutional Review Board.

Inclusion criteria for our analytic sample consisted of children who: (a) were between the ages of 18 to 60 months; (b) received an ASD diagnosis by a licensed, medical provider (e.g., psychiatrist, developmental behavioral pediatrician, or neurodevelopmental pediatrician) or licensed psychologist (clinical or neuro) based on the *Diagnostic and Statistical Manual of Mental Disorders, 5*^th^* Edition* diagnostic criteria (American Psychiatric Association, 2013) and clinical judgment, informed by the *Autism Diagnostic Observation Schedule-Second Edition* (ADOS-2; Lord et al., 2012), medical, developmental and family history, as well as behavioral testing; and (c) completed all pre-appointment paperwork (completion rate = 62%) within 6 months (98% within a week) of their evaluation appointment.

The final analytic sample consisted of 498 autistic children, ranging in age from 18 to 58 months (*M* = 33 months; *SD* = 7 months). Children in this sample were predominantly male (80%), White (45%), and non-Hispanic (93%). 76.5% were diagnosed with ASD by a physician and 23.5% were diagnosed by a licensed psychologist. Parents who completed intake questionnaires and parent-report measures of language and social communication ability consisted of mothers (85%), with a college level education (52%), and private insurance (64%).

### Measures

#### Language and social communication measures for latent profile analysis

Measures selected for the LPA included both parent-report and clinician-administered measures of language and social communication abilities (see Table 2).

**TABLE 1:** Model fit statistics and *n* (%) by class for latent profile models with two to six classes.

						Class *n* (%) based on the estimated model					
No. of classes	AIC	BIC	ICL	Entropy	BLRT	1	2	3	4	5	6
2	16201	16331	16468	0.89	< 0.001	276 (56)	222 (45)				
**3**	**16124**	**16300**	**16461**	**0.83**	**< 0.001**	**237 (48)**	**170 (35)**	**91 (19)**			
**4**	**16073**	**16297**	**16469**	**0.87**	**< 0.001**	**102 (21)**	**173 (35)**	**75 (15)**	**148 (30)**		
5	16114	16383	16548	0.79	0.86	48 (10)	193 (39)	52 (11)	118 (24)	87 (18)	
6	16086	16402	16598	0.76	–	93 (19)	178 (36)	10 (2)	91 (19)	46 (9)	80 (16)

AIC, Akaike Information Criterion; BIC, Bayesian Information Criterion; ICL, Integrated Completed Likelihood; BLRT, parametric bootstrapped likelihood ratio test. Entropy closer to 1 reflects a good classification of participants. A significant BLRT *p*-value indicates that the model with a greater number of classes fit the data better relative to a fewer number of classes.

Bold denotes the two best-fitting models.

**TABLE 2:** Means and standard deviations of language and social communication variables by latent profile.

			Profile 1	Profile 2	Profile 3
Assessment variable	Assessor	Score	(*n* = 237)	(*n* = 170)	(*n* = 91)
ADOS-2 SA CSS (Social Communication)	Clinician	CSS (range = 1–10)	8.10 (1.71)	6.75 (2.14)^a^	6.80 (2.24)^a^
CBCL-Withdrawn (Social Communication)	Parent	T Score (M = 50; SD = 10)	73.7 (10.6)	67.7 (11.1)	64.2 (8.16)
MSEL EL DQ (Expressive Language)	Clinician	DQ (M = 100; SD = 15)	30.5 (10.4)	68.8 (23.9)	54.2 (13.8)
MSEL RL DQ (Receptive Language)	Clinician	DQ (M = 100; SD = 15)	29.1 (9.75)	63.1 (25.1)	54.7 (18.8)
PCL (General Language)	Parent	Composite (range = 0–26)	3.21 (0.67)^a^	7.28 (2.55)	3.27 (0.87)^a^

CSS, Calibrated Severity Score; CBCL, Child Behavior Checklist; MSEL EL DQ, Mullen Scales of Early Learning – Expressive Language Developmental Quotient; MSEL RL DQ, Mullen Scales of Early Learning – Receptive Language Developmental Quotient; PCL, Parent rating of Child Language (Custom score, see text for details).

^a^Denotes non-significant difference between marked groups (*p* > 0.05); All other differences are significant at *p* < 0.05.

##### Parent-rating of child language

During the clinic intake process, parents responded to five yes/no items and two multiple choice items about their child’s receptive, expressive, and pragmatic language abilities on a clinic-specific Parent-rating of Child Language (PCL) questionnaire: (1) Are you worried about your child’s language development? [Yes = 0; No = 1]; (2) Does your child have any problems with talking, hearing, being understood by others, or understanding what he/she is told? [Yes = 0; No = 1]; (3) Can your child speak phrases with at least 2- or 3-word combinations? [Yes = 3; No = 0]; (4) Can your child tell you about their day? [Yes = 3; No = 0]; (5) Can your child have a conversation? [Yes = 5; No = 0]; (6) How does your child usually communicate? [Babbling = 1; Single Words = 2; Short phrases = 3; Full sentences = 4]; (7) Does your child use sign language or any other communication device? [Sign language or PECS or Speech Generating Device = 1; No = 0]. The above codes were derived by weighted parent responses such that a higher code reflected a higher level of language ability. Codes were first created by the first author who is a certified and licensed speech-language pathologist (SLP). The codes were then discussed with and fine-tuned by an interdisciplinary team (which included two additional certified SLPs, two epidemiologists, a neuropsychologist, a psychiatrist, and a developmental behavioral pediatrician). For each participant, scores were summed for a total score ranging from 0 to 18, with higher scores reflecting higher language ability.

##### Mullen scales of early learning: Receptive and expressive language subscales

The Mullen Scales of Early Learning (MSEL; Mullen, 1995) is a clinician-administered standardized developmental assessment for children birth to 68 months. The Receptive Language (RL) and Expressive Language (EL) subscales were administered during the ASD diagnostic evaluation. Consistent with previous literature, developmental quotients (DQs) were calculated by dividing each MSEL subscale age-equivalent score by the child’s chronological age and multiplying by 100 (Messinger et al., 2013). The RL and EL DQs were included in the LPA.

##### Child behavior checklist 1.5-5: Withdrawn subscale

The Child Behavior Checklist 1.5-5 (CBCL; Achenbach and Rescorla, 2000) is a parent-report, norm-referenced reliable and valid questionnaire developed to measure emotional, behavioral, and social limitations in young children. The checklist consists of 99 items describing the presence of a specific behaviors that are rated on a 3-point Likert scale (0 = not true, 1 = somewhat/sometimes true, 2 = very often true). Item scores are summed and converted to T-scores (*M* = 50; *SD* = 10) to derive “problem scores.” The Withdrawn subscale T-score was included in the LPA to capture parent’s ratings of child social communication functioning, as items of this scale include: “avoids looking others in the eye;” “doesn’t answer when people talk to him/her;” “refuses to play active games;” “seems unresponsive to affection;” “shows little affection toward people;” “withdrawn, doesn’t get involved with others.”

##### Autism diagnostic observation schedule, second edition

The Autism Diagnostic Observation Schedule, Second Edition (ADOS-2) is a clinician-administered, semi-structured standardized measure developed to assess the presence or absence of features of ASD related to communication, social interaction, play, and restricted, repetitive behaviors (Lord et al., 2012). The ADOS-2 consists of different modules, with module selection based on chronological age and language ability at the time of testing. Children were administered the ADOS-2 during their ASD diagnostic evaluation. The ADOS-2 was administered by a certified and licensed SLP or a licensed psychologist, clinically trained to administer the ADOS-2. Specifically, all clinicians completed a required ADOS-2 clinical training workshop with a certified ADOS-2 trainer prior to clinical administration of the ADOS-2. Clinicians had access to quarterly booster trainings and research-reliable ADOS-2 clinicians for consultation, if needed. The ADOS-2 Social Affect Calibrated Severity Score (ADOS-2-SA-CSS; score 1 to 10) was derived, reflecting the relative severity of social communication impairment and allowing comparisons across modules (Esler et al., 2015). Higher SA CSS scores reflect greater social communication limitations. The ADOS-2 SA CSS was included in the LPA to capture clinician’s ratings of child social-communication functioning.

#### Correlates of language and social communication profiles

Correlates of language and social communication latent profiles included both sociodemographic and child-based developmental factors hypothesized to account for language and social communication heterogeneity among young autistic children.

##### Sociodemographic factors

Parents completed clinic-specific questionnaires upon initiating their child’s intake process. This form was used for deriving sociodemographic variables. Questionnaires captured the following sociodemographic data: child age, sex, race/ethnicity, parent education, medical insurance, and history of intervention. Race was categorized as a four-level variable (Asian, Black, White, and Other). Other races included Native American, Pacific Islander, multiracial, and any other race. Prior to 2019, ethnicity was captured on this clinic-specific questionnaire as a racial category and therefore informants were unable to report both race and ethnicity for the majority of this study. Parental education was classified as No College Education vs. College Education. Medical Insurance was classified as public (reflecting Medical Assistance) vs. private (e.g., PPO) plans. History of intervention (i.e., parent report of speech therapy or general early intervention services) was derived as a binary variable (yes/no).

##### Auditory processing

Based on the child’s age, auditory processing was measured using the auditory processing subscales from one of five different parent-report questionnaires: the Sensory Processing Measure-Preschool Home Form (SPM-P), the Toddler Sensory Profile-2 (TSP-2), the Child Sensory Profile-2 (CSP-2), the Infant Toddler Sensory Profile (ITSP), or the Short Sensory Profile (SSP). The SPM-P is a 75-item, reliable and valid questionnaire developed to assess seven different sensory processing categories in children aged 2–5 years (Glennon et al., 2011). The TSP-2 and the CSP-2 are part of the Sensory Profile 2, which has high internal consistency, interrater reliability, and test-retest stability (Dunn, 2014). The TSP-2 is a 54-item questionnaire developed to assess seven different sensory processing categories in children 7–35 months old. The CSP-2 is an 86-item questionnaire developed to assess six different sensory processing categories in children aged 3–14 years. The ITSP is a 36-item questionnaire developed to assess six different sensory processing categories. The ITSP has high internal consistency, test-retest reliability, and convergent validity (Dunn, 2002; Ben-Sasson et al., 2008; Beranova et al., 2017; Niedźwiecka et al., 2020). Finally, the SSP is a shortened 38-item version of the Sensory Profile 2 to assess sensory processing in children aged 3–17 years. A binary auditory processing variable was derived such that “typical auditory processing” was defined by a rating of *typical performance* (SPM, ITSP, SSP) or *just like the majority of the others* (CSP, TSP) and “atypical auditory processing” was defined by all other ratings. We did not differentiate between over and under processing since SPM (*Typical, Some Dysfunction, Definite dysfunction*) does not have this distinction and comprised 70% of the data.

##### Non-verbal problem-solving and fine motor skills

The MSEL’s (described above) Visual Reception (VR) and Fine Motor (FM) subscales were administered during the ASD diagnostic evaluation. VR and FM DQs were derived and included as continuous variables in the multinomial logistic regression analysis.

##### Co-occurring features of attention deficit/hyperactivity disorder and anxiety

The CBCL, as described above, yields a total of five DSM-based condition scores. To include measures of ADHD and anxiety traits, respective subscale T-scores were included as continuous variables in the multinomial logistic regression analysis. A T-score of 70 and above reflects “clinically significant” features.

### Statistical analysis

Distinct language and social communication profiles were derived using Latent Profile Analysis (LPA). LPA is a person-centered, mixture modeling approach for detecting and estimating underlying sample clustering. LPA divides participants into distinct subgroups, for the purposes of maximizing within group homogeneity, based on the posterior probability of continuous indicator responses (i.e., phenotypic characteristics). In the current study, parent-reported (PCL and CBCL–Withdrawn T-Score) and clinician-administered (MSEL EL-DQ, RL-DQ, and ADOS SA-CSS) measures served as model indicators. These measures were used to identify distinct language and social communication profiles among young autistic children.

Models with two to six class solutions were fitted to the data and compared in: (a) goodness of fit statistics, (b) proportion of participants classified within each profile, and (c) whether the profiles were clinically meaningful. Fit statistics included the Akaike Information Criterion (AIC), Bayesian information criterion (BIC; Schwarz, 1978), Integrated Completed Likelihood (ICL; Biernacki et al., 2000), Entropy, and Parametric Bootstrapped likelihood ratio test (BLRT; McLachlan and Rathnayake, 2014). Lower values of BIC, AIC and ICL indicate better model fit. Entropy values closer to 1 denote an improvement in the classification of participants. A significant *p*-value associated with the BLRT for a given model indicates an improvement in fit compared to *K*-1 classes. After determining the optimal number of classes, participants were assigned class membership based on their posterior probability. All variables were converted to a z-score distribution (*M* = 0, *SD* = 1), to provide a uniform metric for the LPA. Lower z-scores reflect greater impairment.

After determining class membership, differences in sociodemographic and child-based factors were examined using chi-square tests for categorical measures and one-way analysis of variance for continuous measures. Two separate multinomial regression models–one for sociodemographic characteristics and one for child-based developmental factors–were then used to find adjusted correlates of language and social communication profiles. The outcome for all models was odds of belonging to a particular latent profile and the correlates were those identified as significant (*p* < 0.05) from the bivariate analysis. We opted to use two separate models as we did not have *a priori* hypotheses regarding how different latent profiles and child-based developmental differences would interact with sociodemographic factors. In addition, we found that child-based factors were strongly associated with group membership leading to very little variability that could be explained by sociodemographic factors. Finally, we wanted to avoid potential collinearity between child age and the fine motor and non-verbal problem-solving DQs, as age is part of the calculation of all DQs. Therefore, sociodemographic and child-based factors were examined in separate models to obtain interpretable associations.

All analyses were completed in R Version 1.2.5033 (R Core Team, 2020) using packages mclust (Scrucca et al., 2016) and nnet (Ripley and Venables, 2022). All tests were 2-sided with *p*-values of < 0.05 considered statistically significant.

## Results

### Latent profile analysis

As shown in Table 1, AIC, BIC, and ICL values were the lowest for the 3- and 4-class models. Entropy values for the 3- and 4-class solutions suggested similar classification of participants (3-class entropy = 0.83 and 4-class entropy = 0.87). However, two classes in the 4-class model were nearly identical, with differences only observed on the ADOS SA CSS (6.39 vs. 9.34). As a result, the 4-class solution was deemed less interpretable, and the 3-class model was selected as the more clinically meaningful solution.

**FIGURE 1:**
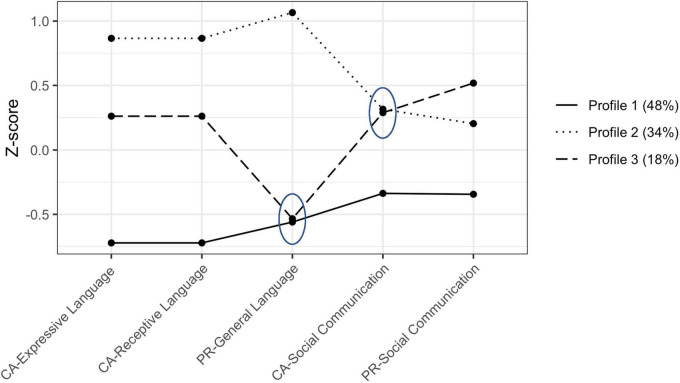
Language and social communication variable z-scored means for the three-profile solution. Ellipses encompass means that are not significantly different. CA, clinician-administered; PR, parent-rated. Profile 1, “Relatively Low Language and Social Communication Abilities;” Profile 2, “Relatively Elevated Language and Social Communication Abilities;” Profile 3, “Informant Discrepant Language and Relatively Elevated Social Communication Abilities.”

Figure 1 shows standardized means by profile and Table 2 shows the actual means by profile.

Profile 1 “Relatively Low Language and Social Communication Abilities”, included 48% of the sample (*n* = 237; 77% male). Children in Profile 1 were characterized by the lowest levels of language and social communication abilities on both clinician-administered and parent-report measures. These children also exhibited the lowest non-verbal problem solving (*M* = 54.1; *SD* = 17.0) and fine-motor skills (*M* = 61.1; *SD* = 14.9) and included the highest percentage of autistic children with atypical auditory processing (67%), and relatively more features of ADHD (*M* = 58.7; *SD* = 7.9) compared to Profile 2.

Profile 2 “Relatively Elevated Language and Social Communication Abilities”, included 34% of the sample (*n* = 170; 82% male). Children in Profile 2 were characterized by the highest levels of language and social communication ability on both clinician and parent reported measures. These children also exhibited the highest non-verbal problem solving skills (*M* = 76.0; *SD* = 22.8), which were significantly elevated compared to Profile 1, and the most features of anxiety (*M* = 56.7; *SD* = 9.81), which was significantly elevated compared to Profile 3, but not Profile 1.

Profile 3 “Informant Discrepant Language and Relatively Elevated Social Communication Abilities”, included 18% of the sample (*n* = 91; 86% male). Children in Profile 3 were characterized by elevated levels of social communication ability on both clinician-administered and parent-report measures but moderate (clinician-administered) and low (parent-report) levels of language ability. These children exhibited the highest fine motor skills (*M* = 78.8; *SD* = 24.5) compared to both Profiles 1 and 2. Only in comparison to Profile 1, Profile 3 included the lowest percentage of children with atypical auditory processing (51%) and fewer features of ADHD (*M* = 56.3.0; *SD* = 7.14). Finally, compared to Profile 2 only, Profile 3 showed significantly fewer features of anxiety (*M* = 54.0; *SD* = 6.01).

### Correlates of language and social communication profiles

Bivariate relationships between latent class membership and sociodemographic and child-based correlates are presented in Table 3. Among the sociodemographic variables, child age, race, parental education and medical insurance status were significantly different (*p* < 0.05) across groups. The multinomial logistic regression included these sociodemographic variables while controlling for child age (Table 4). Children with college-educated parents were more likely to be in Profile 2 (OR = 2.61; *p* < 0.001) and Profile 3 (OR = 1.99; *p* < 0.02), compared to Profile 1. Compared to White children, Black children were less likely to be in Profile 2 (OR = 0.52; *p* = 0.04), relative to Profile 1, and more likely to be in Profile 3 (OR = 2.88; *p* = 0.03) compared to Profile 2. Insurance and other race categories were not significant after adjusting for parent education.

**TABLE 3:** Sociodemographic and child characteristics by latent profile.

N (%)	Profile 1	Profile 2	Profile 3	*P*-value
	237 (48%)	170 (34%)	91 (18%)	
Age at evaluation (*M, SD*)	2.65 (0.58)	3.03 (0.59)	2.46 (0.56)	< 0.001
Male sex (*n*,%)	181 (76.7)	139 (82.2)	78 (85.7)	
Race (*n*,%)				0.02
Asian	9 (3.81)	16 (9.47)	4 (4.40)	
Black	77 (32.6)	31 (18.3)	28 (30.8)	
White	100 (42.4)	86 (50.9)	39 (42.9)	
Other	50 (21.2)	36 (21.3)	20 (22.0)	
Ethnicity (*n*,%)				0.72
Hispanic	18 (7.79)	10 (6.10)	5 (5.68)	
Not Hispanic	213 (92.2)	154 (93.9)	83 (94.3)	
Parent education (*n*,%)				< 0.001
No College Degree	143 (61.1)	54 (32.1)	39 (42.9)	
College Degree	91 (38.9)	114 (67.9)	52 (57.1)	
Medical insurance (*n*,%)				< 0.001
Medicaid/Public	108 (45.8)	40 (23.7)	32 (35.2)	
Private	128 (54.2)	129 (76.3)	59 (64.8)	
History of intervention (*n*,%)	195 (84.8)	145 (85.8)	73 (84.9)	0.96
MSEL VR DQ (*M, SD*)	54.1 (17.0)	76.0 (22.8)	73.5 (17.5)	< 0.001
MSEL FM DQ (*M, SD*)	61.1 (14.9)	73.5 (15.4)	78.8 (24.5)	< 0.001
Auditory processing (*n*,%)				0.02
Atypical	156 (67.0)	96 (57.1)	46 (51.1)	
Typical	77 (33.0)	72 (42.9)	44 (48.9)	
CBCL, ADHD (*M, SD*)	58.7 (7.85)	57.4 (7.91)	56.3 (7.14)	0.03
CBCL, Anxiety (*M, SD*)	55.1 (7.19)	56.7 (9.81)	54.0 (6.01)	0.02

M, mean; SD, standard deviation; MSEL VR DQ, Mullen Scales of Early Learning Visual Reception Developmental Quotient; MSEL FM DQ, Mullen Scales of Early Learning Fine Motor Developmental Quotient; CBCL, Child Behavior Checklist; ADHD, attention deficit/hyperactivity disorder.

**TABLE 4:** Parameter estimates of the sociodemographic factors multinomial logistic regression model.

	Profile 2 vs. 1	Profile 3 vs. 1	Profile 3 vs. 2	Wald	Pairwise
	OR (CI)	OR (CI)	OR (CI)		
**Age at evaluation**	3.53 (2.37–5.26)^c^	0.53(0.33–0.85)^b^	0.12^c^(0.06–0.21)	72.73^c^	2 > 1 > 3
**Race**					
White	REF	REF	REF		
Black	0.52 (0.29–0.93)^a^	1.34 (0.7–2.56)	2.88 (1.28–6.59)^a^	6.42^a^	3 > 2, 1 > 2
Asian	0.89 (0.51–1.56)	0.94 (0.49–1.82)	0.95 (0.43–2.03)	0.08	–
Other	1.48 (0.58–3.77)	0.82 (0.23–2.91)	0.55 (0.13–1.89)	1.23	–
**Parent education**					
No college	REF	REF	REF		
College degree	2.62 (1.59–4.32)^c^	2.00 (1.11–3.58)^c^	1.00 (0.48–2.10)	16.30^c^	2 > 1, 3 > 1
**Medical Insurance**					
Medicaid/Public	REF	REF	REF		
Private	1.46 (0.84–2.55)	1.18 (0.62–2.26)	0.67 (0.29–1.53)	1.74	–

OR = Odds Ratio; CI = Confidence Interval; ^a^*p* < 0.05; ^b^*p* < 0.01; ^c^*p* < 0.001.

As shown in Table 5, the child-based variables associated with group membership were VR DQ (non-verbal problem solving skills), FM DQ (fine motor skills), CBCL ADHD (features of ADHD), CBCL Anxiety (features of Anxiety) and auditory processing. Compared to Profile 1, higher fine motor, non-verbal problem-solving skills, and typical auditory processing were associated with increased likelihood of being in Profile 2 [non-verbal problem solving: odds ratio (OR) = 1.06; auditory processing: OR = 1.99; all *p*s < 0.05] or Profile 3 (non-verbal problem solving: OR = 1.04, fine motor skills: OR = 1.04; auditory processing skills: OR = 2.07; all *p* < 0.05). Higher ADHD symptomatology were significantly associated with lower likelihood of being in Profile 3 (OR = 0.96; *p* = 0.04) but not Profile 2 as compared to Profile 1. Higher non-verbal problem-solving skills (OR = 1.02), lower fine motor skills (OR = 0.96), and higher levels of anxiety (OR = 1.05) were significantly associated with being in Profile 2 compared to Profile 3.

**TABLE 5:** Parameter estimates of the child-based factors multinomial logistic regression model.

	Profile 2 vs. 1	Profile 3 vs. 1	Profile 3 vs. 2	Wald	Pairwise
	OR (CI)	OR (CI)	OR (CI)		
MSEL VR DQ	1.06 (1.04–1.08)^c^	1.04 (1.02–1.06)^c^	0.98 (0.96 – 0.99)^a^	41.63^c^	2 > 3 > 1
MSEL FM DQ	1.01 (0.98–1.03)	1.04 (1.02–1.07)^b^	1.04 (1.01–1.06)^b^	11.93^b^	3 > 1,3 > 2
**Auditory processing**					
Atypical	REF	REF	REF		
Typical	1.99 (1.16–3.41)^a^	2.07 (1.13–3.78)^a^	0.97 (0.53–1.78)	8.08^a^	2 > 1,3 > 1
CBCL, ADHD problems	0.96 (0.93–0.99)^a^	0.97 (0.93–1.01)	1.01 (0.97–1.06)	4.64	1 > 2
CBCL, Anxiety problems	1.04 (0.99–1.07)	0.99 (0.95–1.04)	0.95 (0.91–0.99)^a^	5.88^a^	2 > 3

OR, Odds Ratio; CI, Confidence Interval; ^a^*p* < 0.05; ^b^*p* < 0.01; ^c^*p* < 0.001.

## Discussion

The current study aimed to understand the heterogeneity in language and social communication profiles, and their sociodemographic and child-based developmental correlates, within a large clinic-based sample of young autistic children. Using LPA, a person-centered approach to statistical modeling, three meaningful profiles of language and social communication abilities were identified from parent-report and clinician-administered measures: Profile 1 “Relatively Low Language and Social Communication Abilities”, Profile 2 “Relatively Elevated Language and Social Communication Abilities”, Profile 3 “Informant Discrepant Language and Relatively Elevated Social Communication Abilities.” Slightly less than half of the children were in Profile 1, whereas a third were in Profile 2 and the remaining eighteen percent were in Profile 3. Overall, profiles were distinguished by different levels of language and social communication ability (e.g., high, medium, and low) and discrepant parent-report and clinician measurement of language (i.e., Profile 2 vs. 3). Significant differences were found in the sociodemographic and child-based developmental correlates of these profiles, including level of parental education, race, non-verbal problem-solving skills, fine motor skills, auditory processing, and co-occurring child mental health characteristics (i.e., ADHD and anxiety).

Despite previous reports suggesting that autistic children present with a unique language profile such that use of language (i.e., expressive language) exceeds the ability to understand language (i.e., receptive language; Charman et al., 2003; Luyster et al., 2008; Hudry et al., 2010), we did not observe such a pattern in any of the three profiles identified in the current study. Instead, our results are consistent with findings from a meta-analysis, revealing no specific receptive-expressive profile among young autistic children (Kwok et al., 2015). Taken together, our findings suggest that a receptive-expressive language discrepancy is not a common profile in autistic toddlers or preschoolers. However, differences may emerge as a child develops language, beyond toddlerhood and preschool age, if abilities take divergent trajectories.

Characterizing almost half of the sample, autistic children in Profile 1 exhibited the lowest scores across clinician-administered measures of receptive language, expressive language, and social communication as well as the lowest scores on a parent-rated measure of social communication abilities. Perhaps not surprisingly, children in Profile 1 also exhibited the lowest scores in non-verbal problem-solving, fine motor skills, auditory processing, and high levels of ADHD symptoms. These results are consistent with literature indicating that autistic children with co-occurring ADHD symptoms often have greater impairments in communication and social communication skills (Rao and Landa, 2014; Lyall et al., 2017; Yerys et al., 2019), and consistent with an extant and growing body of evidence suggesting that children with ASD plus high ADHD symptoms tend to present with generally lower developmental functioning (Karalunas et al., 2018; Antshel and Russo, 2019; Miller et al., 2020; Hong et al., 2021; Reetzke et al., 2021a).

Children from the lowest-resource households (defined by lower levels of parent education and a higher percentage of families with a public medical insurance) tended to be in Profile 1. This group also included a higher percentage of Black children, which may be suggestive of potential racial disparity in services. Indeed, recent reports indicate that children from underrepresented racial groups are less likely to receive an ASD evaluation compared to their non-Hispanic White counterparts by three years (Maenner, 2021), which inevitably leads to delays in access to intervention services. However, our findings indicated that there was no significant difference regarding history of intervention for the children in Profile 1. This finding is consistent with reports that early intervention (e.g., Birth-to-3 speech-language therapy) may not be predictive of language growth in autistic children from 2.5 to 5.5 years of age (Ellis Weismer and Kover, 2015). The low level of language, social communication, and developmental functioning may instead reflect the *quality and quantity* of intervention services children in Profile 1 received compared to children in the other groups. Even if these children did receive a comparable quantity of services, which could not be identified given the dichotomous classification, the intervention received may have not met the specific needs of the child and family. Unfortunately, the current data are limited in being able to pinpoint the exact mechanism underlying this disparity. These findings highlight the need for effective community-based implementation strategies for autistic children from low-resource households and underrepresented communities to improve access to individualized, quality care. For example, the Exploration, Preparation, Implementation, Sustainment (EPIS; Aarons et al., 2011), an implementation framework focused on influential contextual factors (e.g., service environment, policies, family cultural characteristics, etc.), can be used in partnership with community stakeholders to identify potential barriers that may hinder the uptake of community-based early interventions (Stahmer et al., 2019).

Children in Profile 2 exhibited relatively elevated language and social communication abilities, as well as non-verbal problem-solving skills and fewer parent-endorsed features of ADHD. Children in this group included a higher percentage of children from White, college-educated families. Children in Profile 2 were uniquely characterized by a higher level of parent-endorsed features of anxiety, consistent with the previous literature showing young autistic children with higher expressive language abilities tend to exhibit higher levels of anxiety symptoms, and vice versa (Davis et al., 2012; Vasa et al., 2016; Rodas et al., 2017). This may be a function of measurement limitations given that most parent-report based measures of anxiety, like the CBCL, are highly reliant on a child’s ability to verbally express their anxiety. Thus, our present findings, and the broader literature, may underestimate the presence of anxiety symptoms in young autistic children until reliable and valid measures are developed for individuals at all levels of language ability (i.e., non-speaking to speaking).

Profile 3 had similarly low parent-rated language abilities as Profile 1, yet relatively moderate language abilities per clinician-administered direct measures of receptive and expressive language, reflecting an informant discrepancy in language abilities. This finding contrasts with Profiles 1 and 2 as well as extant and emerging literature (Miller et al., 2017; Reetzke et al., 2021b), showing that parent report of language abilities does not significantly differ from clinician assessment of receptive and expressive language skills. To better understand why this pattern of informant discrepancy was only observed for Profile 3, we examined whether there were specific language differences between Profiles 3 and 1 which might not have been captured by our clinic-based, parent-report measure of child language.

Parents of 32- to 36-month-old children categorized as Profiles 3 and 1 reported similar concerns about expressive and receptive language skills, indicating that although their children used some single words they were not yet using two- or three-word phrases to communicate. However, parents of children in Profile 1 reported that their children typically babbled to communicate, while parents of children in Profile 3 indicated that their children typically used single words to communicate. While our clinic-based parent-report measure of child language was not sensitive to these differences, the MSEL receptive and expressive language scales were. For example, children in Profile 3 used single words to label objects and pictures in their environment on the MSEL, while children in Profile 1 were not yet able to do this. Overall, these findings highlight the importance of collecting multiple types of parent-report and clinician-administered measures to estimate a child’s language ability during the clinic evaluation process.

In terms of general patterns observed across correlates of language profiles, our finding that children from households with higher parental education were in the relatively elevated language and social communication Profile 2 and children with lower parental education tended to be in the relatively low language and social communication Profile 1 is consistent with the literature showing strong association between parental education and language abilities in autistic children (Anderson et al., 2007; Pungello et al., 2009; Warlaumont et al., 2014; Ellis Weismer and Kover, 2015; Olson et al., 2021). In addition, our findings are aligned with the majority of extant literature which has found a strong positive association between non-verbal cognitive ability and language abilities (Anderson et al., 2007; Wodka et al., 2013; Thurm et al., 2015; Yoder et al., 2015; Brignell et al., 2019). Even after consideration of other correlates of language and social communication abilities, non-verbal problem-solving skills were associated rather robustly with language and social communication profile membership. In addition, consistent with previous findings (Bhat et al., 2012; LeBarton and Iverson, 2013; LeBarton and Landa, 2019; Bal et al., 2020; Iverson, 2021), we found a significant association between fine motor skills and latent profile group membership. Here, Profile 3 showed relative strengths in fine motor skills compared to the other two profiles. These findings provide further support for the role of non-verbal problem-solving and fine motor skills in the development of receptive and expressive language; and highlight the importance of early intervention focused on these developmental domains as potential pathways for improving language outcomes in young autistic children.

### Limitations

The results of this study should be interpreted within the context of several limitations. First, with only one time point, we are unable to examine the temporal stability of the sociodemographic and child-based developmental correlates of language and social communication profiles identified in the current study. Research is needed to replicate the findings of the present study in a longitudinal cohort to examine whether the correlates of language and social communication profiles in young autistic children are predictive of more developmentally downstream language and social communication outcomes. Second, our findings may not be representative of the general autistic population, as our participants were recruited from a single autism specialty clinic, limiting the generalizability of these findings. Third, our clinic-based, parent-rated measure of child language ability was a relatively short, omnibus measure of language ability which differed from our clinician-administered standardized measure of receptive and expressive language ability. Although our parent-rated measure of child language ability was strongly correlated with the MSEL receptive and expressive language subscales, it is possible that our LPA may have yielded different results with more comparable parent-rated and clinician-administered measures of receptive and expressive language ability. Fourth, the measures included in the current study were limited to what was available in the patient medical records (e.g., history of intervention captured as a binary variable), introducing potential measurement bias. Future work should aim to capture both the quantity and quality of intervention services to better understand how disparities in access to intervention services may be associated with variability in language and social communication profiles among young autistic children.

## Conclusion

An LPA identified three language and social communication profiles based on parent-report and clinician-administered measures of language and social communication ability. Children from the lowest-resource households exhibited the lowest language and social-communication abilities, and the lowest non-verbal problem solving and fine-motor skills, along with more features of attention-deficit/hyperactivity disorder and atypical auditory processing. These findings highlight the need for effective community-based implementation strategies for autistic children from low-resource households to improve access to individualized quality care.

## Data availability statement

The original contributions presented in this study are included in the article/supplementary material, further inquiries can be directed to the corresponding author.

## Ethics statement

The studies involving human participants were reviewed and approved by Johns Hopkins Medicine Institutional Review Board. Written informed consent from the participants’ legal guardian/next of kin was not required to participate in this study in accordance with the national legislation and the institutional requirements.

## Author contributions

RR and RL conceived, outlined, and supervised the original study. JH and VS acquired the data. VS organized the database and performed the statistical analyses. RR and VS wrote the first draft of the manuscript. All authors contributed to the refinement of the research questions and methodology, contributed to the interpretation of the results, critical revision of the manuscript, read, and approved the submitted version.
